# Fluorescence anisotropy assays for high throughput screening of compounds binding to lipid II, PBP1b, FtsW and MurJ

**DOI:** 10.1038/s41598-020-63380-2

**Published:** 2020-04-14

**Authors:** Adrien Boes, Samir Olatunji, Tamimount Mohammadi, Eefjan Breukink, Mohammed Terrak

**Affiliations:** 1 InBioS-Centre d’Ingénierie des Protéines, Liège University, B6a, Quartier Agora, allée du six Août 11, 4000 Liège 1, Belgium; 2 Membrane Biochemistry and Biophysics, Department of Chemistry, Faculty of Science, Utrecht University, Utrecht, The Netherlands; 3 Present Address: School of Medicine and School of Biochemistry and Immunology, Trinity College Dublin, DO2 R590 Dublin, Ireland

**Keywords:** Biological techniques, Analytical biochemistry, High-throughput screening, Sensors and probes, Biochemistry, Enzymes, Microbiology, Antimicrobials, Bacteria, Infectious-disease diagnostics

## Abstract

Lipid II precursor and its processing by a flippase and peptidoglycan polymerases are considered key hot spot targets for antibiotics. We have developed a fluorescent anisotropy (FA) assay using a unique and versatile probe (fluorescent lipid II) and monitored direct binding between lipid II and interacting proteins (PBP1b, FtsW and MurJ), as well as between lipid II and interacting antibiotics (vancomycin, nisin, ramoplanin and a small molecule). Competition experiments performed using unlabelled lipid II, four lipid II-binding antibiotics and moenomycin demonstrate that the assay can detect compounds interacting with lipid II or the proteins. These results provide a proof-of-concept for the use of this assay in a high-throughput screening of compounds against all these targets. In addition, the assay constitutes a powerful tool in the study of the mode of action of compounds that interfere with these processes. Interestingly, FA assay with lipid II probe has the advantage over moenomycin based probe to potentially identify compounds that interfere with both donor and acceptor sites of the aPBPs GTase as well as compounds that bind to lipid II. In addition, this assay would allow the screening of compounds against SEDS proteins and MurJ which do not interact with moenomycin.

## Introduction

**Figure 1:**
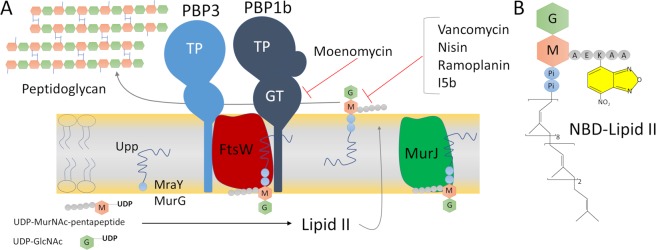
Schematic representation of the late stages of peptidoglycan synthesis. (**A**) The lipid II precursor is assembled at the inner face of cytoplasmic membrane by MraY and MurG from soluble precursors (UDP-MurNAc-pentapeptide and UDP-GlcNAc) and the undecaprenyl-phosphate (Upp) lipid carrier. Lipid II is then transported to the outer face of the membrane by MurJ/FtsW where it is polymerized by FtsW and PBPs. The mode of action of the antibiotics are indicated with red lines. (**B**) Structure of the fluorescent NBD-labeled lipid II. Upp is attached to the disaccharide MurNAc-GlucNAc via a pyrophosphate (Pi-Pi). AEKAA depicts the pentapeptide sequence L-Ala-D-Glu-L-Lys-D-Ala-D-Ala with the NBD fluorophore bound to the Lysin residue.

Most bacteria surround their cytoplasmic membrane with a peptidoglycan (PG) sacculus that protects the cell from bursting due to the turgor and maintains cell shape^1^. In order to propagate, bacteria have to enlarge and divide their cell envelope including their PG sacculus, a net-like polymer consisting of glycan strands made of alternating β-1,4-linked *N*-acetylglucosamine (Glc*N*Ac) and *N*-acetylmuramic acid (Mur*N*Ac) residues cross-linked by peptides^1^. The substrate for PG synthesis is the lipid II precursor (undecaprenyl-pyrophosphoryl-MurNAc(pentapeptide)-(beta-1,4)-GlcNAc), that is synthesized on the inner face of the cytoplasmic membrane and subsequently translocated through the membrane by a flippase (MurJ/FtsW)^2–4^. MurJ is a member of the multidrug/oligosaccharidyl-lipid/polysaccharide (MOP) exporter superfamily^5^. RodA and FtsW of the SEDS (shape, elongation, division, and sporulation) family were found to have glycosyltransferase activity whose function in case of FtsW depends on a cognate class B penicillin-binding proteins (bPBP)^6,7^. When on the periplasmic side of the membrane, lipid II is polymerized by the GTase activities of the class A PBPs (aPBPs) and SEDS proteins (Fig. 1). The resulting glycan chains are cross-linked by the transpeptidase (TPase) activities of aPBPs and bPBPs^6–9^ (Fig. 1). The functions of these proteins are coordinated with those of interacting proteins in the elongasome and divisome complexes to ensure optimal growth and division of the bacterial cell^10–13^.

Because of their essential role in PG synthesis, the lipid II substrate, its transport across the cytoplasmic membrane (MurJ/FtsW) and its polymerization into PG (PBPs and SEDS) are key target candidates for new antibacterial discoveries. It is not surprising that in their warfare against bacteria, microorganisms (including bacteria) have developed an array of molecules and strategies that inhibit cell wall PG assembly at different stages^14,15^. The most well-known antibiotics are beta-lactams that covalently bind to the PBPs/TPase active sites and block peptide cross-linking leading to cell lysis. A second strategy is the inhibition of glycan chains polymerization catalysed by the glycosytransferases of family 51 (GT51 domains of aPBPs and monofunctional GTases)^9^. The enzymatic cavity of these proteins can be divided into two substrate binding sites: a donor site for the lipid-bound growing glycan chain, that is also the moenomycin A binding site, and an acceptor site for lipid II. Moenomycin A, is the only known natural product that specifically binds to the GT51 active sites and competitively inhibits PG synthesis^16^. It is a potent antibiotic that is not used in human therapy but was used in animal feedstock for decades without reported resistance^17^. Bacteriophages also target PG for progeny release, a small *Escherichia coli* bacteriophage levivirus M was found to encode a protein antibiotic (Lys^M^) that specifically inhibits the activity of MurJ and induces lysis of the host^15^. Many other cell wall inhibitors such as the natural products vancomycin, ramoplanin, teixobactin and nisin, bind directly to the lipid II precursor by different mechanisms and prevent its access and further processing by proteins interacting with lipid II^14,18,19^.

The fight against antimicrobial resistance requires the exploration of new bacterial targets to identify novel antibiotic classes. In this regard, the peptidoglycan biosynthesis pathway, a proven excellent target of the most prescribed drugs (β-lactam antibiotics) used in the treatment of bacterial infections, is still not fully explored as new essential components and molecular mechanisms are being discovered (MurJ and SEDS proteins).

In this work, we have developed a fluorescent anisotropy assay (also known as fluorescence polarization assay) using a unique and versatile probe (nitrobenzoxadiazole (NBD)-labelled lipid II) to monitor binding between lipid II and interacting proteins (PBP1b, MurJ and FtsW), as well as between lipid II and interacting antibiotics (vancomycin, nisin, ramoplanin and a small molecule). Competition experiments are also shown as proof-of-concept for the use of this assay in a high throughput screening of compounds against all these targets. In addition, the method also represents a powerful tool for the study of the mode of action of compounds that interfere with these processes.

## Results

### Interaction of NBD-lipid II with PBP1b, FtsW and MurJ using FA assay

**Figure 2:**
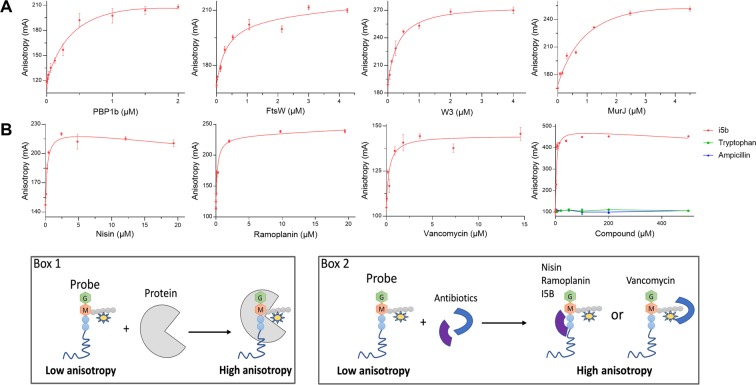
Fluorescence anisotropy assay using the NBD-lipid II as a probe for binding studies with proteins and antibiotics. FA (in mA units) is plotted as a function of protein or antibiotic concentrations. The error bars represent the FA values as mean ± s.d. of triplicate experiments using the same buffer and protein conditions. (**A**) Direct binding of NBD-lipid II probe (0.33 µM) to PBP1b, FtsW, FtsW-PBP3 complex and MurJ. (**B**) Direct binding of NBD-lipid II probe (0.33 µM) to different antibiotics (nisin, ramoplanin, vancomycin, and the small molecule I5b. The last panel also shows that tryptophan and ampicillin used as controls do not bind to the probe. Box 1 and Box 2 schematically illustrates the corresponding FA assay and the observed FA variations.

**Figure 3:**
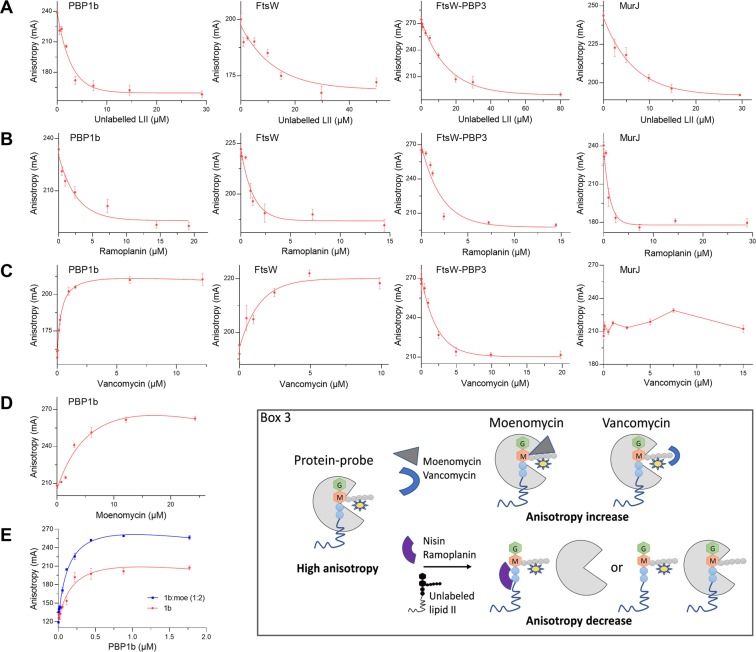
Competition assays of unlabelled lipid II and antibiotics for the proteins (PBP1b, FtsW, FtsW-PBP3 and MurJ)/NBD-lipid II binding show FA modifications (increase or decrease) of the probe. FA (in mA units) is plotted as a function of compound concentrations. The error bars represent the FA values as mean ± s.d. of triplicate experiments using the same buffer and protein conditions. (**A,B**) Competitive displacement of the NBD-lipid II probe from binding proteins by unlabelled lipid II and ramoplanin. (**C**) The increase of FA with vancomycin indicates the formation of a ternary complex with PBP1b:probe and FtsW:probe; the decrease of FA in the presence of vancomycin indicate competitive displacement of the probe from FtsW-PBP3; the addition of vancomycin to MurJ:probe does not induce significant modification of the FA. (**D**) Binding of the probe to PBP1b in the presence of increasing moenomycin concentrations, moenomycin induces an increase of FA indicating the formation of a ternary complex with PBP1b:probe. (**E**) Moenomycin binding to PBP1b (PBP1b:moenomycin,1:2 molar ratio) induces and increase the FA compared to PBP1b alone. Box 3 schematically illustrates the corresponding FA assays and the observed FA variations.

To develop a fluorescence anisotropy (FA) assay for interaction studies between lipid II and lipid II-binding proteins or antibiotics, we attached the NBD fluorophore to the lysin residue of the peptide moiety to generate the fluorescent probe NBD-lipid II (Fig. 1B). Three known lipid II-interacting proteins from *E. coli* (Fig. 1A), PBP1b, FtsW (and the complex FtsW-PBP3) and MurJ, have been purified to study their interaction with NBD-lipid II using a FA assay **(**Fig. S1**)**. Before the binding studies, the optimal conditions for each protein and concentration of the probe were determined. We have also verified that PG polymerization by PBP1b was insignificant in the absence of divalent cations (FA assay condition) (Fig. S2). The concentration of the probe was set at 0.33 µM and incubated with increasing concentrations of each protein. The results show an increase of the FA signal as a function of protein concentrations until saturation, the signal dynamic range ΔmA was ~100 (Fig. 2A). The three proteins and the complex bind NBD-lipid II with high affinity and the determined *K*_*d*_ values were between 0.3 and 1.1 µM (Table S1). FtsN used as control had no effect on the FA of the probe (Fig. S3). Interestingly, the binding of fluorescent NBD-lipid II to PBP1b, MurJ, FtsW and FtsW-PBP3 could be displaced by increasing concentrations of unlabelled lipid II (Fig. 3A, Table S1), which has no direct effect on the FA of the probe (Fig. S4). These results provide a proof-of-concept that the assays could be used for screening of compounds interfering with the probe (substrate) binding to the target proteins. It is worth noting that the *K*_*d*_ value of the probe for PBP1b (0.5 ± 0.2 µM) is in the same micromolar range as the *K*_*m*_ value (~2 µM). The *K*_*d*_ values of the probe for FtsW and FtsW-PBP3 are similar (0.3 ± 0.1 µM), indicating that PBP3 does not significantly contribute to lipid II binding. The *K*_*d*_ value for FtsW is ~4 time lower compared to that of MurJ (1.1 ± 0.3 µM), indicating that in these conditions FtsW has a higher affinity for lipid II than MurJ. These results are consistent with our previous finding^13^ but, are different from those observed by native mass spectrometry, a *K*_*d*_ value of 2.9 µM was determined for lipid II binding to MurJ and the *K*_*d*_ value for FtsW was higher and could not be determined by this technique^20^.

### Interaction of NBD-lipid II with antibiotics using FA assay

Four antibiotics known to interact with lipid II, vancomycin, nisin, ramoplanin and a small molecule (I5b) have been selected to study their direct interactions with NBD-lipid II using the FA assay. The incubation of the probe with increasing concentrations of the lantibiotic nisin (MM 3354 Da), the glycopeptide vancomycin (MM 1449 Da) and the glycolipodepsipeptide ramoplanin (MM 2254 Da) induced an increase of FA (Fig. 2B). In contrast, moenomycin A (MM 1582 Da), and unlabeled lipid II (MM 1876 Da), used as non-binder controls and with molecular masses similar to those of vancomycin and ramoplanin, did not significantly affect the FA signal of the NBD-lipid II probe (Fig. S4). We have previously discovered a small molecule called I5b that inhibits peptidoglycan polymerization through binding to a phosphate group of lipid II^21^. Interestingly, here we show that it is possible to monitor I5b-binding to NBD-lipid II using the FA assay (Fig. 2B). Ampicillin or tryptophan (indole group is present in the I5b compound) used as a small molecule controls have no significant effect on the FA of the probe (Fig. 2B). These results show that our FA assay can accurately detect interactions between various classes of antibiotics that interact with lipid II by different mechanisms.

### Competition of antibiotics with NBD-lipid II:proteins using FA assay

For the completion assays, we choose the lipid II-binding antibiotics used above (nisin, vancomycin, ramoplanin and the small molecule I5b). As a model of protein-binding antibiotic, we used moenomycin which binds to the donor site of the GTase domain of aPBPs and MGTs and inhibits their polymerase activity. The proteins, PBP1b, MurJ, FtsW and FtsW-PBP3 complexes and NBD-lipid II were mixed with increasing concentrations of the antibiotics. Nisin and ramoplanin both bind to the pyrophosphate moiety of lipid II. They induced FA signal decreases with all proteins, indicating that both antibiotics were able to prevent the formation of the respective NBD-lipid II:protein complexes by sequestrating lipid II (Figs. 3B and S5). This is consistent with the published result obtained by native mass spectrometry for lipid II:MurJ with ramoplanin^20^. In contrast, the addition of vancomycin to PBP1b or FtsW and NBD-lipid II mixture induced an increase of the FA signal in both cases (Fig. 3C). These results indicate that vancomycin binding to the D-Ala-D-Ala moiety does not prevent NBD-lipid II binding to PBP1b and FtsW (conversely probe binding to PBP1b or FtsW does not prevent vancomycin binding) but only prevents its processing by these enzymes. Thus, the ternary complexes, NBD-lipid II:PBP1b:vancomycin and NBD-lipid II:FtsW:vancomycin could be formed, this has been observed previously for FtsW^3^. These results demonstrate that our FA assay is able to discriminate between the modes of action of nisin and ramoplanin and that of vancomycin. Notably, when FtsW-PBP3 complexed with the probe was used instead of FtsW alone, vancomycin induced a decrease in the FA signal, showing that vancomycin was able to compete with FtsW-PBP3 for lipid II binding (Fig. 3B). This result probably reflects the accessibility of the D-Ala-D-Ala motif in a way that vancomycin was able to displace lipid II from FtsW-PBP3 (without necessarily changing the affinity for lipid II). This is consistent with the previous observation showing that PBP3 binding to FtsW facilitates the accessibility of lipid II by PBP1b^13^, presumably through conformational changes of FtsW induced by PBP3. This is also consistent with the stimulation of the GTase activity of FtsW by a cognate bPBP observed with several SEDS-bPBP pairs^7^. As a consequence, to take advantage of this property, the screening of compounds could be performed with the FtsW-PBP3 complex (or other SEDS-bPBP complexes), that showed similar behaviors in the FA assay with all the compounds tested, instead of FtsW alone. Titration of MurJ and the probe by vancomycin does not induce a significant change of the FA (Fig. 3C), suggesting that the peptide moiety was not accessible to vancomycin in our conditions, while a ternary complex MurJ:lipid II:vancomycin was previously reported using native mass spectrometry^20^.

Moenomycin binds to the donor site of the GTase domain of PBP1b and has no effect on the FA of the probe (Fig. S4). Thus, we expected that binding of moenomycin to the donor site will displace the probe from this site and induce a decrease of the FA. Surprisingly, the FA increased with increasing moenomycin concentrations (Fig. 3D). Similarly, comparison of the binding of NBD-lipid II to the PBP1b or PBP1b-complexed with moenomycin (protein:moenomycin molar ratio 1:2) shows a higher FA signal for PBP1b:moenomycin when compared to PBP1b alone (Fig. 3E). This result can be explained on the basis of our previous finding showing a positive cooperativity between the donor and acceptor sites of the GTase domain^22^. Indeed, using surface plasmon resonance we showed that the binding of lipid II or analogs to the acceptor site of *Staphylococcus aureus* MtgA facilitates the binding of moenomycin to the donor site by allosteric effect. Thus, the results indicate that moenomycin binding to the donor site also increases the affinity of lipid II to the acceptor site of PBP1b GTase domain and confirm the cooperativity mechanism between the donor and acceptor sites of these enzymes. These experiments reveal the versatility of the FA technique: in addition to its ability to identify compounds binding to the active site of PBP1b and potentially to other GT51 enzymes, it also allows the study of their complex mechanism.

## Discussions

The final stages of peptidoglycan assembly include the translocation of the lipid II precursor by MurJ/FtsW from the inner to the outer side of the cytoplasmic membrane and its polymerization by the PBPs and SEDS transglycosylases. These processes are essential for peptidoglycan sacculus growth and division and constitute important hot spot targets for known and new antibiotics that can interfere with them. In addition, the lipid II precursor itself is a target of many antibiotics such as vancomycin, ramoplanin or teixobactin. Therefore, an assay based on lipid II as a probe, has the advantage to identify new antibiotics that bind to lipid II or to the proteins allowing its translocation or polymerization into peptidoglycan.

Biochemical production of unlabelled and labelled lipid II, although still a tedious process, becomes feasible in high yield, mainly thanks to the accessibility of high amounts of the limiting undecaprenyl phosphate moiety, which can be isolated from plant leaves^23^. This prompted us to develop a FA assay, based on NBD-lipid II as a probe for use in the identification of compounds that interact with lipid II or interfere with its enzymatic processing. We have estimated that 0.9 µmole of labelled-lipid II could allow the screening of 100,000 compounds using our standard FA assay. Based on our preliminary data, further optimization and miniaturization could reduce the required amount of the probe by a third.

The FA assay described here allows on the one hand, estimation of direct binding of fluorescent-lipid II to interacting proteins (PBP1b, FtsW and MurJ) and on the other hand, the accurate detection of various classes of antibiotics that bind lipid II by different mechanisms. Interestingly, direct interaction of lipid II with both FtsW and MurJ could be detected, using the same technique for the first time, which is consistent with their proposed biochemical function (whatever their exact function, they must interact with lipid II) while other techniques can detect lipid II interaction with only one protein^13,20^. This show that the FA assay is highly sensitive and preserves the stability of the proteins.

Competition experiments performed using unlabelled lipid II, four lipid II-binding antibiotics (vancomycin, nisin, ramoplanin and I5b) and moenomycin demonstrate that the assay can detect compounds interacting with the probe or the proteins even without completely dissociating the complex. The latter case results in an increase of the FA as observed in the experiments with vancomycin and moenomycin. This shows that an inhibitory compound could either increase or decrease the FA signal and therefore compounds that induce significant FA variation in the competition assay should be considered as potential hits and further tested for lipid II probe binding without protein and activity-based assays. In addition to the ability of the FA assay to identify new inhibitors of the PG assembly, it could also constitute a valuable tool in inferring the mechanism of action and mode of binding of new compounds by comparisons with known inhibitors. As the lipid II probe is also the substrate of the flippase and the PG polymerases, the FA assay would also allow the study of the molecular mechanisms of these enzymes and their complexes as observed here with the effect of PBP3 on FtsW and that of moenomycin on PBP1b.

All together the results show that a FA assay based on fluorescent lipid II would allow the identification of new lipid II-interacting antibiotics among both natural products and small molecules libraries as well as compounds binding to the target proteins. FA is a widely used technique in drug research and easily transposable to high-throughput format. Importantly, our FA assay has the advantage, over a previous FA assay based on fluorescent moenomycin, to potentially identify compounds that interfere with both donor and acceptor sites of the aPBPs GTase as well as compounds that bind to lipid II. In addition, this assay would allow the screening of compounds against the SEDS proteins (or SEDS-bPBPs) and MurJ which do not interact with moenomycin.

## Materiel and methods

### Reagents

The synthesis and purification of the cell wall precursor Nitrobenzoxadiazole (NBD)-labelled Lipid II (probe) and unlabelled lipid II (undecaprenyl-pyrophosphoryl-MurNAc(pentapeptide)-(beta-1,4)-GlcNAc) was performed as described^24,25^. The NBD fluorophore is attached to the lysin residue of the peptide moiety (L-Ala-D-Glu-L-Lys-D-Ala-D-Ala) (Fig. 1).

Nisin was obtained from Handary (Brussels), vancomycin was purchased from Sigma and ramoplanin from Abcam. Moenomycin A was a gift from Aventis.

### Expression and purification of proteins

The proteins used in this work were expressed and purified as previously described: PBP1b^26^, FtsW, FtsW-PBP3, MurJ^13^, and FtsN^12^. Stock proteins (1 mg/ml) were stored at −20 °C in the following buffers: PBP1b (25 mM Tris–HCl pH 7.5, 0.5 M NaCl, 0.7% CHAPS). FtsW (50 mM Hepes pH 7.5, 0.3 M NaCl, 5% glycerol, 0.05% DDM). FtsW-PBP3 (50 mM Hepes pH 7.5, 0.3 M NaCl, 10% glycerol, 0.05% DDM). MurJ (20 mM Hepes pH 7.5, 20 mM NaCl, 0.24% DM). FtsN (25 mM Tris–HCl pH 7.5, 0.5 M NaCl, 10% glycerol, DDM 0,05%). The buffer conditions in the FA-assays (final concentration) were as follow: PBP1b in 25 mM Tris–HCl pH 7.5, 0.1 M NaCl, 0.14% CHAPS. FtsW and FtsW-PBP3 in 50 mM Hepes pH 7.5, 0.1 M NaCl, 0.017% DDM (or 0.005% DDM, 0.2% CHAPS) and 5% glycerol. MurJ in 20 mM Hepes pH 7.5, 20 mM NaCl, 0.12% DM (or 0.08% DM, 0.2% CHAPS). FtsN in 25 mM Tris-HCl pH 7,5, 0.017% DDM, 0.1 M NaCl, 10% glycerol.

### Fluorescent anisotropy assay

#### Proteins:NBD-lipid II interaction

Fluorescent anisotropy **(**FA) experiments were performed in a black 384-well plate (Greiner Bio-One Polystyrene Non-Binding Flat Bottom) in triplicates. Serial dilutions of the protein (PBP1bγ, FtsW, FtsW-PBP3 or MurJ) in the appropriate buffer and the NBD-lipid II probe was added at a 0.33 µM final concentration. FtsN was used as a negative control under the same conditions. Blank samples without proteins were also prepared for background fluorescence estimation. The mixtures (30 µL) were allowed to reach equilibrium for 20 min at room temperature with gentle shaking and the FA signals were recorded at 21 °C using an Infinite F Plex (Tecan, Männedorf, Switzerland) microplate reader equipped with polarization filters with excitation and emission wavelengths at 485 nm and 535 nm respectively. Fluorescence polarization (FP) and anisotropy (*A*) values were calculated using the equations *FP* = (*I*_*||*_ − *G·I*_┴_*)/I*_*||*+_ *G·I*_┴_) *and A* = *2· FP/3-FP respectively*, where *I*_*||*_ is the fluorescence intensity of emitted light parallel to excitation, *I*_┴_ is the fluorescence intensity of emitted light perpendicular to excitation, and *G* is the correction factor that correct for instrument bias. The *G* factor is experimentally determined using the probe alone. FA in millianisotropy units (mA) is plotted as a function protein concentration in µM. For *K*_*d*_ determination, the fluorescence anisotropy data were analysed by nonlinear curve fitting to the Eq. (1) using GraphPad Prism 6.0 software^27^.

1 $$(A-{A}_{min})/({A}_{max}-{A}_{min})=[RL]=({K}_{d}+[R]+[L])-{[({K}_{d}+R]+[L])}^{2}-4[R][L]{]}^{1/2}$$

Where *A, A*_*min*_*, A*_*max*_ are the observed, the minimum and the maximum anisotropy, respectively, and [R], [L] and [RL] are the equilibrium concentration of the protein, the probe; and the protein-probe complex, respectively; *K*_*d*_ is the dissociation constant between the protein and the probe.

The fluorescence intensities of bound and free species were evaluated to verify the influence of binding partners on the fluorescence intensity of the probe (Fig S6). Except from ramoplanin and compound 5b, the binding of the proteins or the compounds have no significant effect on the fluorescence intensities (within the experimental error). For these compounds, the anisotropy values were corrected (*Ac*) using Eq. (2) ^28^:

2 $$Ac=\frac{[(A-Af)/(Ab-A)]\cdot (Qf/Qb)(Ab)]+Af}{1+[(A-Af)/(Ab-A)(Qf/Qb)]}$$

where *A* is the observed anisotropy; *A*_*f*_ is the anisotropy of the free probe; *A*_*b*_ is the anisotropy of the probe in the bound state; *Q*_*f*_ is the intensity of free probe and *Q*_*b*_ is the intensity of the probe in the bound state.

#### NBD-lipid II – compounds interaction

Serial dilutions of each antibiotic in 10 mM Tris-HCl pH 7.5 and 3% DMSO were prepared in 384-well plates (30 µL) in triplicates. The probe NBD-lipid II was used at a 0.33 µM final concentration. Blank samples without antibiotic were also prepared for background fluorescence estimation. The mixtures (30 µL) were allowed to reach equilibrium for 20 min at room temperature with gentle shaking and the FA signals were recorded at 21 °C using an Infinite F Plex (Tecan, Männedorf, Switzerland) microplate reader equipped with polarization filters with excitation and emission wavelengths at 485 nm and 535 nm respectively. The data were analysed as described above with [R] and [RL] are, respectively, the equilibrium concentration of the compound and the compound-probe complex.

#### Competition assay

Fixed concentrations of NBD-lipid II (0.33 µM) and proteins (~1 µM, 50–80% FA saturation^29^) were used. Serial dilutions of each antibiotic,unlabelled lipid II, or control compounds were prepared in 384-well plates in triplicates. Blank samples without proteins were also prepared for background fluorescence estimation. The mixtures (30 µL) were allowed to reach equilibrium for 20 min at room temperature with gentle shaking and the FA signals were recorded at 21 °C using an Infinite F Plex (Tecan, Männedorf, Switzerland) microplate reader equipped with polarization filters with excitation and emission wavelengths at 485 nm and 535 nm respectively. The data were plotted FA in millianisotropy units (mA) as a function of test compound concentration in µM. For the analysis of the completion data, fluorescence anisotropy values were fitted to the Eq. (3) using GraphPad Prism 6.0 software^30^.

3 $$[RL]=[R]/[1+{K}_{d}/[L](1+[C]/){K}_{i}]$$

Where [*RL*] is concentration of the receptor-probe complex; [*R*] is the concentration of the protein; [*L*] is the concentration of the probe; [*C*] is the concentration of test compound; *K*_*d*_ is the dissociation constant between the receptor and the probe (determined above); *K*_*i*_ is dissociation constant between the test compound and its target.

### Continuous GTase activity fluorescence assay

#### Standard assay

The PBP1b activity assays with dansyl-lipid II or NBD-lipid II as substrate were performed in a medium binding black 96-well microplate at 30 °C (Greiner Bio One) as described^13,31^. The samples contained 10 µM fluorescent lipid II, 50 mM HEPES-NaOH pH 7.5, 200 mM NaCl, 10 mM CaCl_2_, 0.085% of decyl-PEG, 20% of dimethylsulfoxide (DMSO) and 1 unit of *Streptomyces globisporus N*-acetylmuramidase (Sigma). The reactions were initiated by the addition of 100 nM PBP1b and monitored by following the fluorescence decrease over 20 min at 30 °C using an Infinite 200 PRO Microplate reader (Tecan, Männedorf, Switzerland) with excitation and emission wavelengths at 340 nm and 520 nm respectively for dansyl and with excitation and emission wavelengths at 485 nm and 535 nm respectively for NBD.

#### Modified assay (without divalent citations)

To test the activity of PBP1b in the conditions used in the FA assay, the buffer composition of the standard assay was replaced by the following mixture: 25 mM Tris–HCl pH 7.5, 0.1 M NaCl, 0.14% w/v CHAPS, 10% DMSO.

## Supplementary information

Supplementary Information.
