# Supplementary material for: Fluorescence anisotropy assays for high throughput screening of compounds binding to lipid II, PBP1b, FtsW and MurJ
Source: Sci Rep. 2020 Apr 14;10:6280. doi: 10.1038/s41598-020-63380-2 (PMC7156629; doi:10.1038/s41598-020-63380-2)
Supplement: Supplementary file 1 — Supplementary Information. [file 41598_2020_63380_MOESM1_ESM.pdf]

Supplementary information

Fluorescence anisotropy assays for high throughput screening of compounds binding to lipid II, PBP1b, FtsW and MurJ

Adrien Boes[1], Samir Olatunji[1#], Tamimount Mohammadi[2], Eefjan Breukink[2] and Mohammed Terrak[1]

[1] InBioS-Centre d'Ingénierie des Protéines, Liège University, B6a, Quartier Agora, allée du six Août 11, 4000 Liège 1, Belgium

[2] Membrane Biochemistry and Biophysics, Department of Chemistry, Faculty of Science, Utrecht University, The Netherlands.

[#] present address: School of Medicine and School of Biochemistry and Immunology, Trinity College Dublin DO2 R590, Ireland

To whom correspondence should be addressed:

Mohammed Terrak, Centre d'Ingénierie des Protéines, University of Liège, B6a, Quartier Agora, Allée du six Août 11, 4000 Liège 1, Belgium, Tel.: +33-4366-3332; E-mail: mterrak@uliege.be

Table S1. Binding parameters of NBD-lipid II probe with proteins and antibiotics and K_i values for the displacement of the probe by unlabelled lipid II obtained by FA experiments.

**Protein or antibiotic**	$\boldsymbol{K_d}$ **values (NBD-Lipid II)**	$\boldsymbol{K_i}$ **values (unlabelled-Lipid II)**	**Values from the literature (Lipid II)**
PBP1b	0.5 ± 0.2 µM	1.4 ± 0.2 µM	K_m = 1.8 ± 0.8 µM[1]
FtsW	0.3 ± 0.1 µM	5.9 ± 1.8 µM	*nf*
FtsW-PBP3	0.3 ± 0.1 µM	6.8 ± 1.0 µM	*nf*
MurJ	1.1 ± 0.3 µM	4.6 ± 0.8 µM	K_d = 2.9 ± 0.6 µM [2]
Vancomycin	0.3 ± 0.1 µM	*nd*	K_d = 0.5 µM [3]
Nisin	0.3 ± 0.1 µM	*nd*	K_d 14.6-50 nM [4]
Ramoplanin	0.23 ± 0.05 µM	*nd*	IC_{50} = 0.3 µM [5], K_d = 10-100 nM [6]
I5b	2.8 ± 0.6 µM	*nd*	IC_{50} = 20-56 µM [7]

nd, not determined; *nf* not found

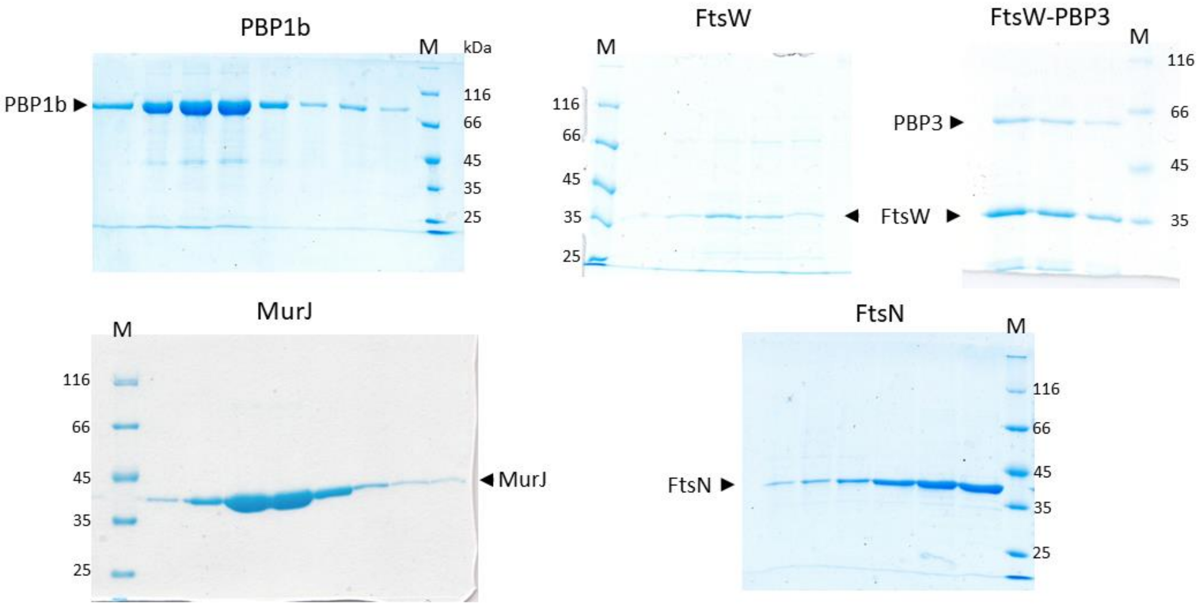

Figure S1. SDS-PAGE showing the purified membrane proteins used in this study. M, molecular mass standard. Calculated molecular masses (MM): PBP1b 90.9 kDa; FtsW 46.7 kDa; PBP3 63.9 kDa; MurJ, 55.7 kDa; FtsN 47 kDa. Membrane proteins mobility in SDS-PAGE is faster than expected from calculated values.

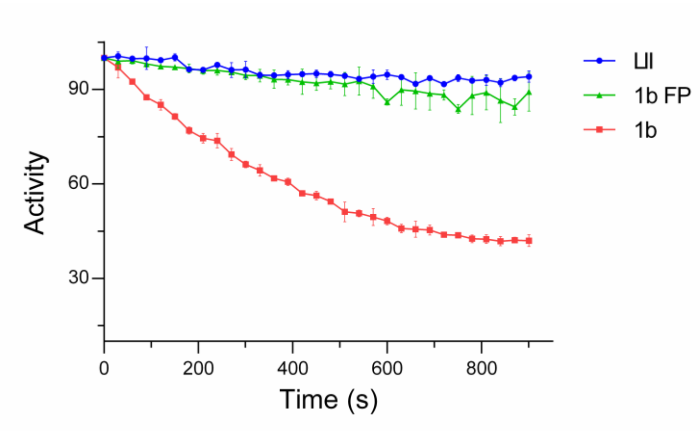

Figure S2. Comparison of the GTase activity of PBP1b (1b) using continuous fluorescence assay in standard condition (presence of Ca^{++}) and in the condition used in the fluorescence anisotropy (FA) experiments (absence of divalent cations). LII depict the fluorescent lipid II substrate without protein. The error bars represent the activity as mean ± s.d. of triplicate experiments.

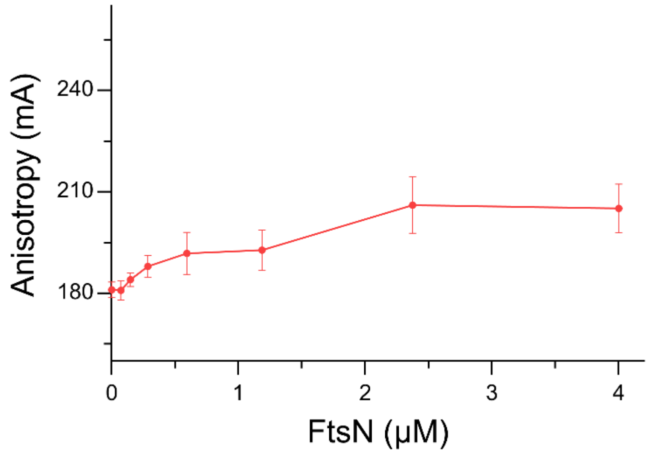

Figure S3. Fluorescence anisotropy assay using FtsN as control. FA (in mA units) is plotted as a function of FtsN concentrations. The error bars represent the FA values as mean ± s.d. of triplicate experiments.

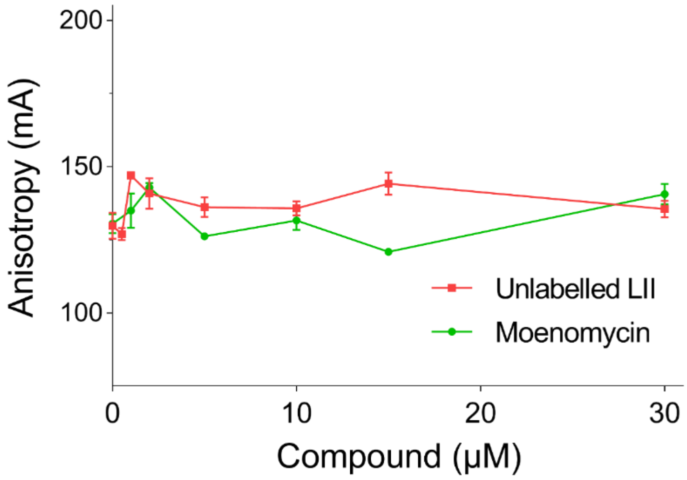

Figure S4. Fluorescence anisotropy assay with unlabelled lipid II (LII) and moenomycin A (moeno) used as controls. FA (in mA units) is plotted as a function of compound concentrations. The error bars represent the FA values as mean ± s.d. of triplicate experiments.

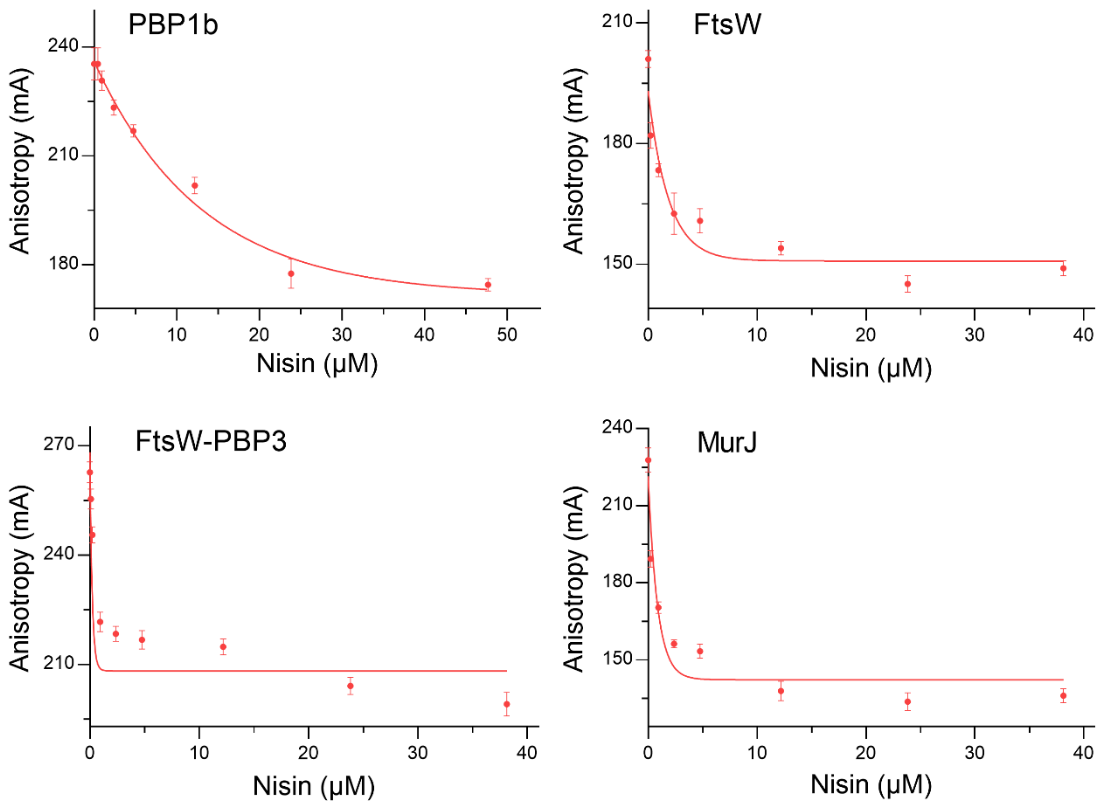

Figure S5. Competitive displacement of the NBD-lipid II probe from binding proteins (PBP1b, FtsW, FtsW-PBP3 and MurJ) by nisin. FA (in mA units) is plotted as a function of nisin concentrations. The error bars represent the FA values as mean ± s.d. of triplicate experiments.

A

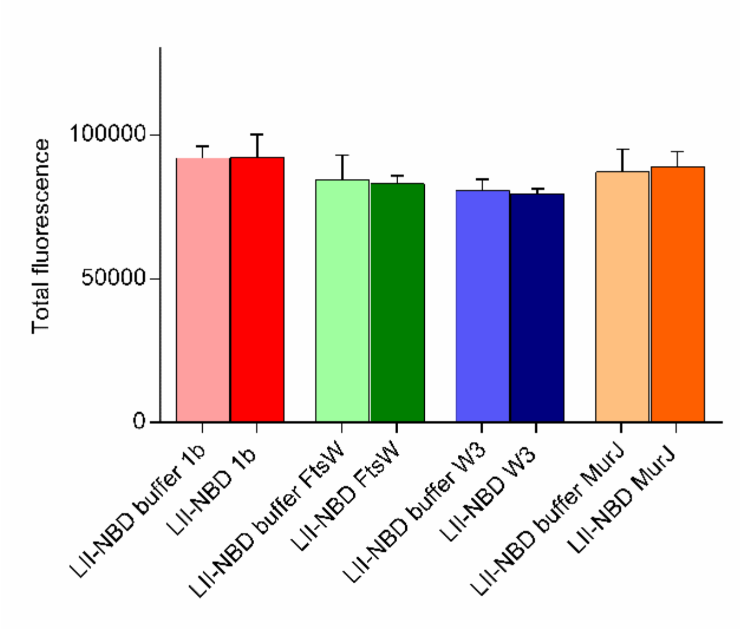

B

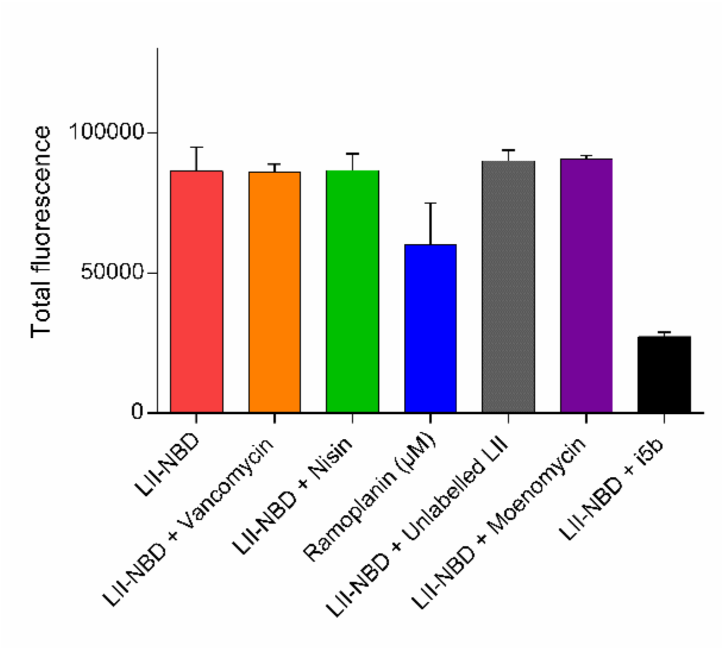

Figure S6. Comparison, under the same conditions, of the fluorescence intensities of probe NBD-Lipid II, LII-NBD) in the free state and bound states with different binding partners (A, Proteins, B, antibiotics or unlabelled lipid II) at saturating concentrations. The error bars represent the FA values as mean ± s.d. of triplicate experiments.

Supplementary references

1. Terrak, M. *et al.* The catalytic, glycosyl transferase and acyl transferase modules of the cell wall peptidoglycan-polymerizing penicillin-binding protein 1b of Escherichia coli. *Mol. Microbiol.* **34**, (1999).
2. Bolla, J. R. *et al.* Direct observation of the influence of cardiolipin and antibiotics on lipid II binding to MurJ. *Nat. Chem.* **10**, 363–371 (2018).
3. Münch, D. & Sahl, H.-G. Structural variations of the cell wall precursor lipid II in Gram-positive bacteria - Impact on binding and efficacy of antimicrobial peptides. *Biochim. Biophys. Acta* **1848**, 3062–71 (2015).
4. 'T Hart, P., Oppedijk, S. F., Breukink, E. & Martin, N. I. New Insights into Nisin's Antibacterial Mechanism Revealed by Binding Studies with Synthetic Lipid II Analogues. *Biochemistry* **55**, 232–237 (2016).
5. Lo, M. C. *et al.* A new mechanism of action proposed for ramoplanin [10]. *Journal of the American Chemical Society* **122**, 3540–3541 (2000).
6. Hu, Y., Helm, J. S., Chen, L., Ye, X. Y. & Walker, S. Ramoplanin inhibits bacterial transglycosylases by binding as a dimer to lipid II. *J. Am. Chem. Soc.* **125**, 8736–8737 (2003).
7. Derouaux, A. *et al.* Small molecule inhibitors of peptidoglycan synthesis targeting the lipid II precursor. *Biochem. Pharmacol.* **81**, 1098–105 (2011).
